# Sex differences in traumatic stress reactivity in rats with and without a history of alcohol drinking

**DOI:** 10.1186/s13293-020-00303-w

**Published:** 2020-05-11

**Authors:** Lucas Albrechet-Souza, Connor L. Schratz, Nicholas W. Gilpin

**Affiliations:** 1 Department of Physiology, School of Medicine, Louisiana State University Health Sciences Center, New Orleans, LA USA; 2 Alcohol & Drug Center of Excellence, School of Medicine, Louisiana State University Health Sciences Center, New Orleans, LA USA; 3 Neuroscience Center of Excellence, School of Medicine, Louisiana State University Health Sciences Center, New Orleans, LA USA; 4 Southeast Louisiana Veterans Health Care System (SLVHCS), New Orleans, LA USA

**Keywords:** Sex differences, Predator odor, Bobcat urine, Alcohol, Stress, Trauma, Startle, Arousal, Corticosterone

## Abstract

**Background:**

Alcohol misuse and post-traumatic stress disorder (PTSD) are highly comorbid, and treatment outcomes are worse in individuals with both conditions. Although more men report experiencing traumatic events than women, the lifetime prevalence of PTSD is twice as high in females. Despite these data trends in humans, preclinical studies of traumatic stress reactivity have been performed almost exclusively in male animals.

**Methods:**

This study was designed to examine sex differences in traumatic stress reactivity in alcohol-naive rats (experiment 1) and rats given intermittent access to 20% ethanol in a 2-bottle choice paradigm for 5 weeks (experiment 2). Animals were exposed to predator odor (bobcat urine) and tested for contextual avoidance 24 h later; unstressed controls were never exposed to predator odor. We evaluated changes in physiological arousal using the acoustic startle response (ASR) test at day 2 post-stress and anxiety-like behavior measured in the elevated plus-maze (EPM) at day 17 post-stress. In experiment 3, time course of corticosterone response was examined in male and female rats following exposure to predator odor stress.

**Results:**

Alcohol-naive males and females exposed to predator odor displayed blunted weight gain 24 h post-stress, but only a subset of stressed animals exhibited avoidance behavior. In alcohol-drinking animals, the proportion of avoiders was higher in males than females, and predator odor exposure increased ASR in males only. Stressed females exhibited blunted ASR relative to unstressed females and stressed males, regardless of alcohol drinking history. Alcohol-experienced females presented lower anxiety-like behavior and higher general activity in the EPM in comparison with alcohol-experienced males. Plasma corticosterone levels were higher in females immediately after predator odor exposure until 60 min post-stress relative to males.

**Conclusions:**

We report robust sex differences in behavioral and endocrine responses to bobcat urine exposure in adult Wistar rats. Also, males with a history of chronic moderate alcohol drinking exhibited increased traumatic stress reactivity relative to alcohol-drinking females. Our findings emphasize the importance of considering sex as a biological variable in the investigation of traumatic stress effects on physiology and behavior.

## Background

Post-traumatic stress disorder (PTSD) is a chronic psychiatric disease that is seen in some but not all individuals after experiencing a traumatic event. Major diagnostic criteria for PTSD include re-experiencing the traumatic event, negative affective state, exaggerated startle responses, and persistent avoidance of trauma-related cues [1]. Women are twice as likely to develop PTSD after trauma [2, 3], and women with trauma exposure and/or PTSD exhibit more sensitivity to and less tolerance of negative emotions [4, 5].

Alcohol use disorder (AUD) is one of the most common co-occurring conditions among individuals diagnosed with PTSD [6, 7]. Approximately one third of individuals with lifetime PTSD also meet the criteria for AUD [8]. Some populations, such as military personnel, are at high risk for AUD and PTSD comorbidities. For example, in Iraq and Afghanistan veterans, 63% of those diagnosed with AUD also met the criteria for PTSD [9]. Although men have a higher prevalence of AUD than women, and women have a higher prevalence of PTSD than men, any individual with either disorder is more likely to have the other [10].

Exaggerated startle response is considered a hallmark symptom of PTSD [1] and is predictive of disease severity in combat-exposed veterans returning home from active duty [11]. In a sample of predominantly male (76.8%) military service members referred for psychiatric evaluation for suicide-related concerns, the hyperarousal symptom cluster was the only significant predictor of subsequent suicide attempts [12]. Although elevated autonomic responses to startling tones have been reported in male and female trauma survivors [13–15], investigations of startle responsivity in patients with PTSD have produced mix results. Male Gulf War veterans with PTSD exhibited exaggerated startle compared to non-PTSD veterans [16]. Conversely, male Vietnam veterans with PTSD did not show increased startle [17] unless they were subjected to a stressful environment [18]. Interestingly, studies in either all [19] or mostly female (66.7%) humans with PTSD [20] report blunted motor responses to acoustic stimuli, suggesting a potential sex difference in traumatic stress effects on acoustic startle responding.

Preclinical studies using animal models to recapitulate PTSD-like behavioral deficits allow investigation of the biological mechanisms underlying traumatic stress effects, but most preclinical research has been conducted in male animals, potentially neglecting issues specific to female subjects [21, 22]. Predator exposure and predator scent are ethologically relevant stressors commonly used as animal models of PTSD. Rodents are exposed to predator odor (PO) in a variety of ways, including cloths containing odor (cat), urine (bobcat, fox), feces or litter, or trimethylthiazoline (TMT)—a synthetic compound isolated from fox feces [23–26]. Although predator stress has been shown to elicit lasting increases in freezing and avoidance behaviors [27, 28], the effects of PO stress on acoustic startle response (ASR) are not consistent. For example, male rats exhibit potentiated ASR magnitude *during* exposure to TMT [29], but three exposures to cat odor (10 min per day separated by a 48-h inter-exposure interval) failed to promote persistent changes on startle reactivity [30].

The current experiments were designed to explore sex differences in traumatic stress reactivity in rats exposed to a PO stress model using bobcat urine developed in our laboratory [31]. The urine of carnivorous species contains 2-phenylethylamine, a trace amine produced by the breakdown of the amino acid phenylalanine [32] that activates trace amine-associated receptor 4 in the rodent olfactory cortex and produces avoidance behavior in rodents [32]. In experiment 1, we tested how male and female rats respond to bobcat urine exposure on a variety of behavioral paradigms including context avoidance, acoustic startle, and the elevated plus-maze (EPM) test. In experiment 2, we evaluated whether stress responsivity to PO changes following chronic voluntary alcohol consumption. Finally, in experiment 3, we examined sex effects on the time course of corticosterone response following exposure to bobcat urine in alcohol-naive male and female rats.

## Methods

### Animals

Eight-week-old male and female Wistar rats (Charles River, Raleigh, NC) were housed in same-sex pairs in a humidity- and temperature-controlled (22 °C) vivarium on a 12-h reversed light-dark cycle (lights off at 7 AM). Animals had ad libitum access to food and water throughout the experiments and were handled daily for 3 min for 1 week before the initiation of experimental protocols. Female rats were freely cycling and assigned to treatment groups without regard to estrous cycle stage.

### Study design

**Fig. 1:**
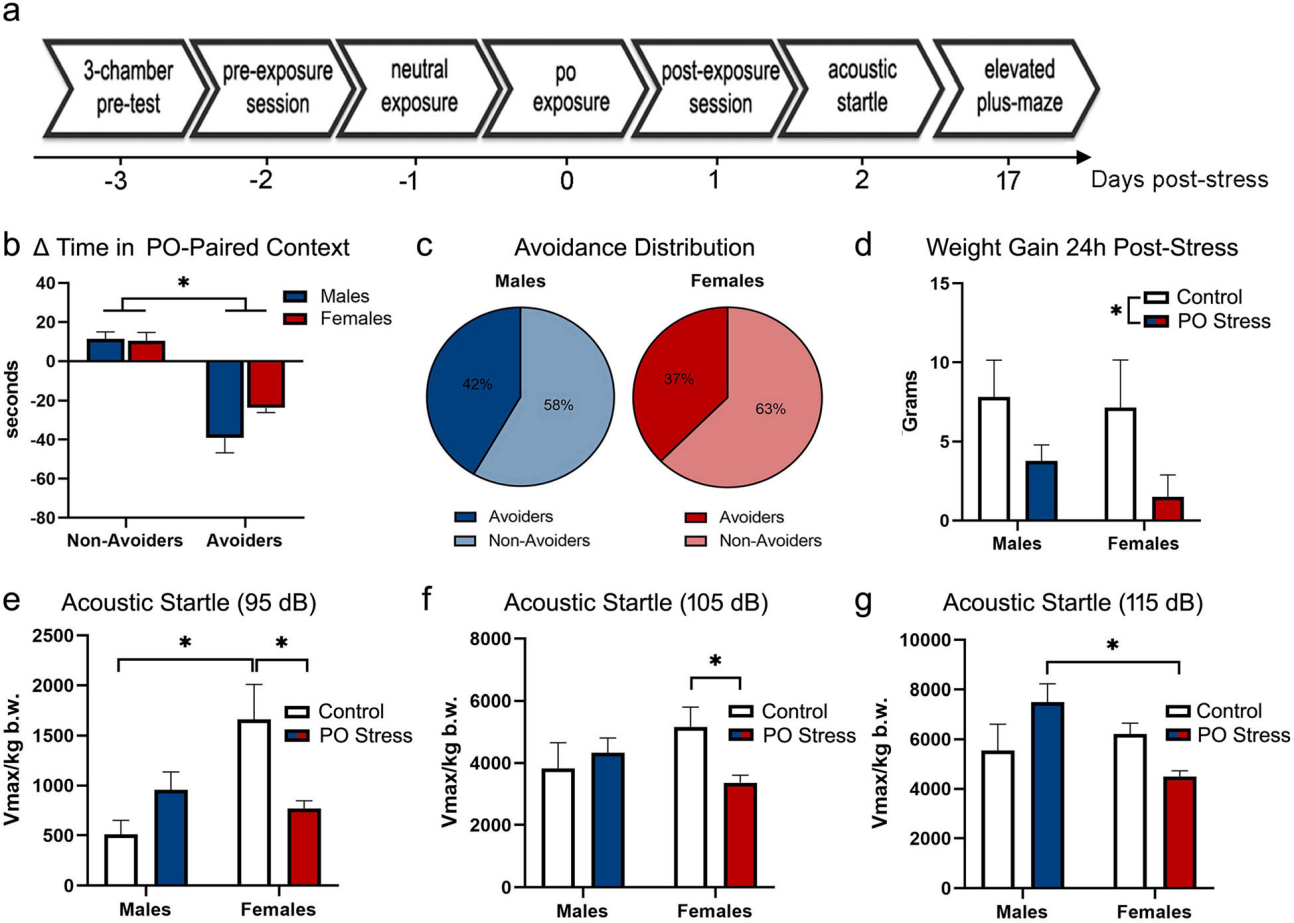
Traumatic stress response in alcohol-naive male and female rats. **a** Experimental design. Rats underwent the conditioned place aversion paradigm using bobcat urine. Controls were never exposed to predator odor (PO) stress. Avoidance behavior was measured 24 h post-stress (day 1). Acoustic startle reactivity was evaluated at day 2 post-stress and is expressed as Vmax normalized by body weight in kilograms (Vmax/kg b.w.). Anxiety-like behavior was tested at day 17 after PO exposure. **b** Change in time spent in PO-paired chamber in rats indexed as avoiders or non-avoiders. **c** Avoidance distribution of stressed rats (avoiders × non-avoiders). **d** Weight gain measured 24 h after exposure to PO. **e** Acoustic startle response at 95 dB. **f** Acoustic startle response at 105 dB. **g** Acoustic startle response at 115 dB. Data presented as mean ± SEM. Asterisk denotes *P* < .05

**Fig. 2:**
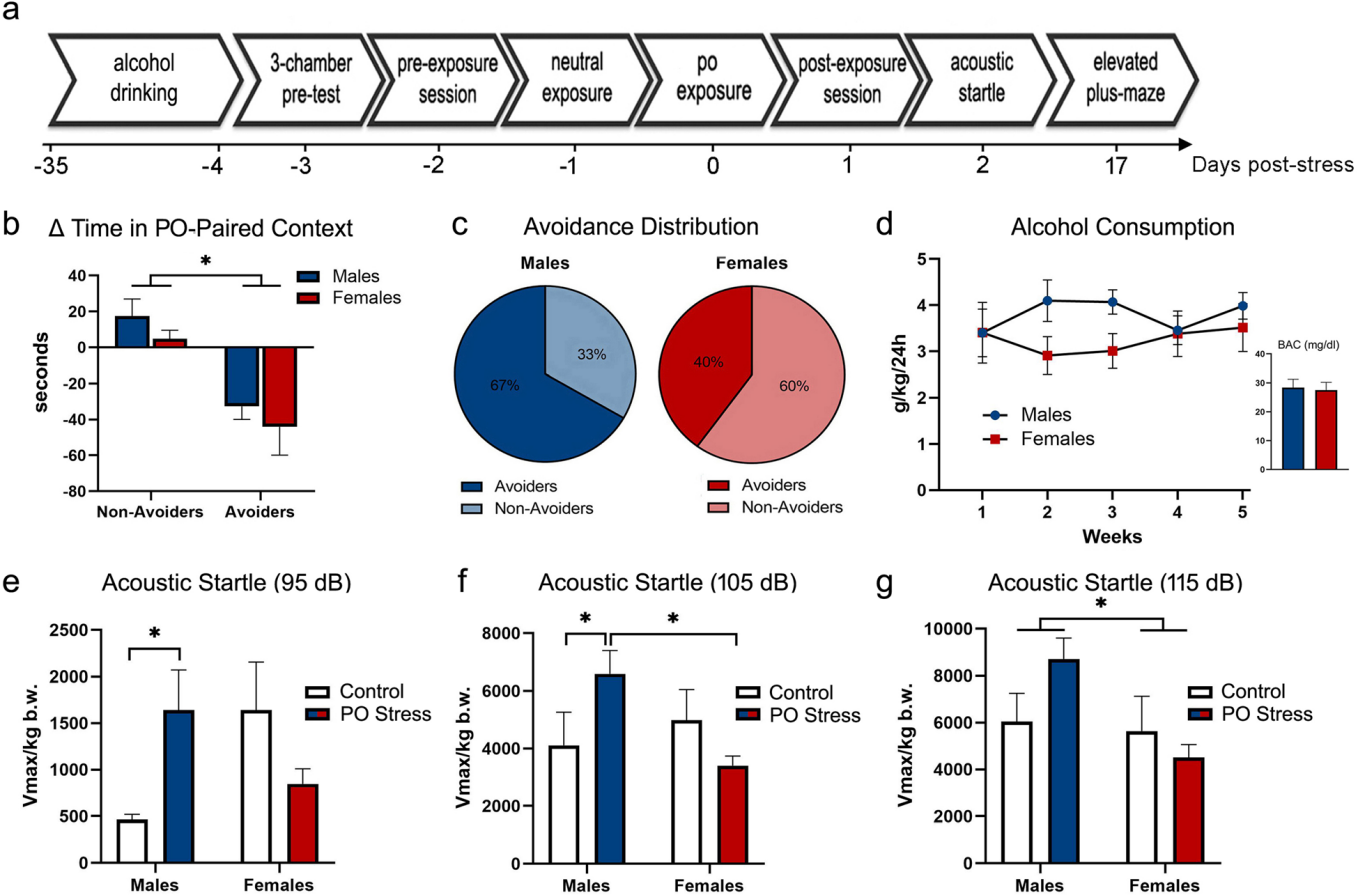
Traumatic stress response in alcohol-drinking male and female rats. **a** Experimental design. Rats were given intermittent access to 20% ethanol in a 2-bottle choice paradigm for 5 weeks followed by conditioned place aversion using bobcat urine. Controls were never exposed to predator odor (PO) stress. Avoidance behavior was measured 24 h post-stress (day 1). Acoustic startle reactivity was evaluated at day 2 post-stress and is expressed as Vmax normalized by body weight in kilograms (Vmax/kg b.w.). Anxiety-like behavior was tested at day 17 after PO exposure. **b** Change in time spent in PO-paired chamber in rats indexed as avoiders or non-avoiders. **c** Avoidance distribution of stressed rats (avoiders × non-avoiders). **d** Alcohol consumption presented as grams of ethanol per body weight in kilograms per rat (estimated from the pair of rats) in 24 h. Insert: blood alcohol concentrations at 2 h after the start of alcohol access during the last drinking session. **e** Acoustic startle response at 95 dB. **f** Acoustic startle response at 105 dB. **g** Acoustic startle response at 115 dB. Data presented as mean ± SEM. Asterisk denotes *P* < .05

**Fig. 3:**
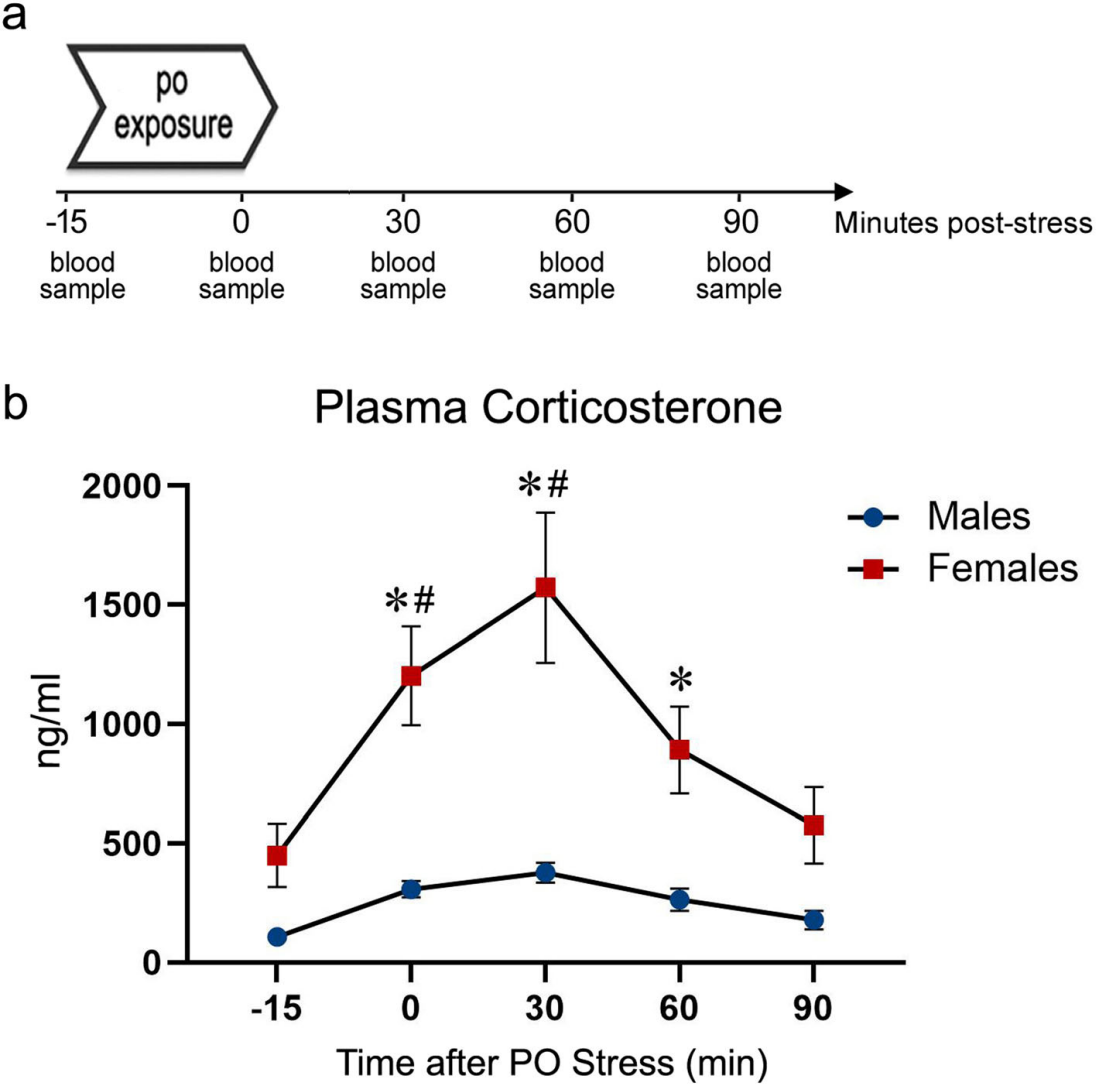
Plasma corticosterone response in male and female rats following exposure to predator odor (PO) stress. **a** Experimental design. Rats were exposed to bobcat urine in a clean cage. Tail blood samples were collected before exposure to PO, immediately after, 30 min, 60 min, and 90 min post-stress. **b** Plasma corticosterone concentrations at the indicated time points. Data presented as mean ± SEM. Asterisk denotes *P* < .05 between sexes; number sign denotes *P* < .05 in comparison with the baseline (− 15 min time point)

In experiment 1, rats were exposed to bobcat urine and tested for avoidance of the odor-paired context 24 h later (*n* = 24 males and 24 females); unstressed controls were never exposed to PO (*n* = 8 males and 8 females). ASR was assessed at day 2 post-stress, and anxiety-like behavior was evaluated at day 17 post-stress using the EPM (Fig. 1a). In experiment 2, pair-housed rats were given intermittent access to 20% ethanol in a 2-bottle choice paradigm for 5 weeks before exposure to bobcat urine. Rats underwent the behavioral test battery as described in experiment 1 (Fig. 2a). This experiment involved 4 groups: control males (*n* = 6), control females (*n* = 6), stressed males (*n* = 12), and stressed females (*n* = 10). In experiment 3 (*n* = 6 males and 6 females), rats were exposed to bobcat urine in a clean cage. Tail blood samples for corticosterone analyses were collected before exposure to PO, immediately after, 30 min, 60 min, and 90 min post-stress (Fig. 3a). These experiments were conducted with independent cohort of rats. Animals were tested in the morning between 9:00 AM and 12:00 PM. At the end of the experiments, rats were euthanized by decapitation under isoflurane anesthesia.

### Predator odor stress

Rats were tested in a 5-day conditioned place aversion procedure [31] that began after the acclimatization period in alcohol-naive rats (experiment 1) or 24 h after the last drinking session in alcohol-experienced rats (experiment 2). On the first day (day -3, Figs. 1a and 2a), rats were allowed 5 min of free exploration of the apparatus (3-chamber pre-test session), which consisted of three large chambers (36 cm length × 30 cm width × 34 cm height) with different types of floor texture (circles, grid, or rod floor) and patterned walls (circles, white, or stripes), separated by a small triangular connecting chamber. The apparatus was thoroughly cleaned between animals with Quatricide® PV in water at a concentration of 1:64 (Pharmacal Research Labs, Waterbury, CT). For each rat, the chamber that exhibited the most deviant time score of the three (i.e., highly preferred or highly avoided) was excluded from all future sessions for that rat. On the next day (day -2), the rat was allowed 5 min to explore the two non-excluded conditioning chambers (pre-exposure session). Rats were assigned to PO stress or unstressed control groups that were counterbalanced for the magnitude of baseline preference for one chamber versus the other (i.e., groups were assigned such that mean pre-existing preference for each of the two chambers was approximately zero for PO stress and control groups). For rats in the PO stress group, an unbiased and counterbalanced design was used to determine which chamber (i.e., more preferred or less preferred) would be paired with PO for each rat. On the next day (day -1), each rat was placed in one of the two chambers with the guillotine door shut without odor for 15 min (neutral exposure). On day 0, rats were placed in the other chamber with the guillotine door shut and a sponge soaked with 3 ml of bobcat urine (*Lynx rufus*; Maine Outdoor Solutions, Hermon, ME) placed under the floor for 15 min (odor exposure). Control rats were treated identically to PO-exposed rats, but the sponges did not contain bobcat urine. On day 1, rats were allowed to explore the two chambers for 5 min (post-exposure session). All testing was conducted under indirect dim illumination (one 60 W white light facing the wall providing approximately 10 lx in the apparatus), and all sessions were recorded and time spent in each chamber was scored by a treatment-blind observer. Avoidance was quantified as a difference score calculated as time spent in odor-paired chamber on day 1 minus time spent in the same chamber on day -2. Rats that displayed > 10-s decrease in time spent in the PO-paired context were classified as avoiders; all other bobcat urine-exposed rats were classified as non-avoiders.

### Intermittent access 2-bottle choice alcohol homecage drinking

Pair-housed adult male and female Wistar rats were given intermittent access to alcohol and water in three 24-h sessions per week for 5 weeks prior to PO stress [33]. We chose this housing condition to avoid exposing animals to social isolation stress. Briefly, rats were weighed and given access to 1 bottle of 20% v/v ethanol and 1 bottle of water approximately 3 h after the start of the dark cycle on Mondays, Wednesdays, and Fridays. After 24 h, the alcohol bottle was replaced with a second water bottle that was available for the next 24 h. Over the weekends, rats had unlimited access to 2 water bottles after the alcohol bottle was removed on Saturday. Bottles were weighed 24 h after alcohol presentation. The position of the alcohol bottle was alternated across sessions to control for side preferences. At the last drinking session, tail blood was collected 2 h after the start of alcohol access to determine blood alcohol concentration (BAC). Rats were restrained manually without anesthesia on a clean surface, and a cut was made 1 mm from the tip of the tail using a razor blade [34]. Blood samples were collected in 1.5 ml microtube and centrifuged at 1900×*g* for 14 min, after which plasma was extracted and immediately analyzed using an Analox AM1 analyzer (Analox Instruments). Alcohol-naive rats (experiment 1) were submitted to the same blood collection procedure 24 h before starting the 5-day conditioned place aversion procedure.

### Acoustic startle response test

At day 2 after exposure to PO stress, rats were tested for acoustic startle reactivity. Rats were placed in a Plexiglas tube attached to an accelerometer inside a dark, soundproof chamber (SR-Lab, San Diego Instruments, CA) and allowed to acclimate for 5 min (75-dB background noise) before the test session [35]. This background white noise was present throughout the session. The chamber and Plexiglas tube were cleaned with Quatricide between each animal. Before testing, an S-R calibrator tube was used to calibrate the chambers. The test session consisted of 30 trials with startle stimuli of three different decibel levels: a 750-ms burst of 95 dB, 105 dB, or 115 dB white noise was randomly presented 10 times each, separated by a 30-s fixed inter-trial interval. The maximum startle response (Vmax, arbitrary units) was recorded during the first 100 ms of each trial.

### Elevated plus-maze test

The EPM test was used to test locomotor and anxiety-like behavior at day 17 after exposure to bobcat urine. The EPM was a black Plexiglas apparatus consisting of two closed arms (50 cm × 10 cm × 40 cm) and two open arms (50 cm × 10 cm) attached to metal legs elevating the maze 50 cm above the ground. All testing was conducted under dim illumination (approximately 10 lx in the open arms). Rats were placed individually in the center of the maze facing a closed arm and allowed 5 min of free exploration. Behavior was recorded with a video camera positioned above the maze. The EPM was cleaned thoroughly between subjects using Quatricide. Video scoring was done by an observer blind to the conditions; we measured percent time spent in the open arms ((time open/time open + time closed) × 100), percent entries in the open arms ((entries open/entries open + entries closed) × 100), and number of closed arm entries. One arm entry was defined as all four paws entering the arm.

### Plasma corticosterone response

Rats were individually transferred from the homecage to a clean cage in a separate room and exposed to bobcat urine for 15 min. Bobcat urine (3 ml) was added to a sponge that was placed beside the cage. Rats were returned to the homecage after PO exposure. Tail blood was collected in EDTA-covered tubes, as described earlier for BAC, at 5 time points: before exposure to PO, immediately after exposure, 30 min, 60 min, and 90 min post-stress. Samples were centrifuged at 1900×*g* for 20 min. Plasma was stored at − 80 °C and analyzed in duplicate for total corticosterone levels using a DetectX ELISA kit (Arbor Assays, Ann Arbor, MI) according to the manufacturer’s instructions.

### Statistical analysis

Data are reported as mean ± SEM, except where otherwise indicated. All statistics were run using Prism 8 (GraphPad, La Jolla, CA). Alcohol-naive (experiment 1) and alcohol-drinking animals (experiment 2) were not tested in parallel; therefore, they are not analyzed together. Measures of Vmax were normalized by body weight in kilograms. Two-way analysis of variance (ANOVA) was performed to analyze change in time spent in PO-paired chamber, body weight gain, activity in the EPM (percent time in the open arms, percent entries in the open arms, and closed arms entries), and startle reactivity at each decibel level—the variables in all cases were sex and stress condition. Dose of alcohol intake (grams of ethanol per kilogram of body weight per rat, estimated from the pair of rats) is presented as the daily average across 3 measurement days per week. Alcohol consumption and plasma corticosterone were analyzed with two-way repeated measure ANOVAs—the variables were sex and sessions. Fisher’s exact test was used to analyze the proportion of avoiders and non-avoiders in each sex. Student’s unpaired *t* test was used to compare the magnitude of avoidance in male and female avoiders*.* A priori Student’s unpaired *t* tests were used to compare the startle response at each decibel level and activity in the EPM (percent time in the open arms, percent entries in the open arms and closed arms entries) between avoiders and non-avoiders in each sex to determine whether they could be collapsed for subsequent analysis. In cases of significant ANOVA effects, post hoc comparisons were performed using Tukey’s multiple comparisons test. Values of *P* < .05 were considered statistically significant.

## Results

### Experiment 1: predator odor stress reactivity in alcohol-naive rats

The timeline of experiment 1 procedure is shown in Fig. 1a. Independent of sex, avoiders exhibited significantly greater avoidance of the PO-paired chamber at 24 h post-exposure (*F* (1, 44) = 78.51, *P* < .0001) relative to non-avoiders (Fig. 1b). There was no significant difference in the magnitude of avoidance between male and female avoiders (Fig. 1b; *t* = 1.86, *P* = .08), and the proportion of animals that met the avoider criteria was similar in both sexes (Fig. 1c; *P* > .05). Likewise, both male and female rats exposed to PO exhibited significantly reduced body weight gain relative to unstressed controls 24 h post-stress (Fig. 1d; stress effect: *F* (1, 44) = 6.99, *P* = .01) but not 4 days post-stress (stress effect: *F* (1, 44) = 1.12, *P* = .29; data not shown).

Because we did not find significant differences in the ASR between avoider and non-avoider males (95 dB: *t* = .61, *P* = .54; 105 dB: *t* = .20, *P* = .84; 115 dB: *t* = .92, *P* = .36) nor between avoider and non-avoider females (95 dB: *t* = .59, *P* = .56; 105 dB: *t* = .45, *P* = .65; 115 dB: *t* = .55, *P* = .59), animals exposed to bobcat urine were pooled into a group designated PO stress and compared to unstressed controls, segregated by sex. To determine whether PO stress affected startle reactivity differently in male and female rats, ASR data were analyzed for each decibel. At 95 dB (Fig. 1e), we found a significant main effect of sex (*F* (1, 58) = 5.45, *P* = .02) and a sex × stress interaction effect (*F* (1, 58) = 10.59, *P* = .002). Tukey’s post hoc comparisons revealed that control females exhibited higher startle reactivity than control males (*P* = .01) and that PO stressed females showed lower startle reactivity relative to control females (*P* = .01). At 105 dB (Fig. 1f), we found a significant sex × stress interaction effect (*F* (1, 58) = 4.11, *P* = .047). Tukey’s post hoc comparison revealed that PO stressed females exhibited lower startle reactivity than control females (*P* = .02). At 115 dB (Fig. 1g), we found a significant sex × stress interaction effect (*F* (1, 58) = 6.02, *P* = .02). Tukey’s post hoc comparison revealed that PO stressed males exhibited higher startle reactivity than PO stressed females (*P* = .0008).

At day 17 post-stress, rats were tested for locomotor activity and anxiety-like behavior in the EPM. Again, we did not find significant differences between avoider and non-avoider males nor between avoider and non-avoider females on percent time in the open arms (males: *t* = 1.58, *P* = .13; females: *t* = 1.25, *P* = .22), percent entries in the open arms (males: *t* = 1.38, *P* = .18; females: *t* = .87, *P* = .39), and closed arms entries (males: *t* = .80, *P* = .43; females: *t* = 1.06, *P* = .30); thus, animals exposed to bobcat urine were pooled into a group designated PO stress and compared to unstressed controls, segregated by sex. There was no significant effect of sex (*F* (1, 59) = .73, *P* = .39) or stress (*F* (1, 59) = .04, *P* = .84) on percent time in the open arms of the EPM (Table 1). Similarly, we did not find significant effect of sex (*F* (1, 59) = .08, *P* = .78) or stress (*F* (1, 59) = .009, *P* = .92) on percent entries in the open arms of the EPM (Table 1). General locomotor performance was assessed by counting closed arm entries in the EPM. Neither sex (*F* (1, 59) = .59, *P* = .45) nor stress (*F* (1, 59) = 3.65, *P* = .06) affected the number of closed arms entries (Table 1).

**Table 1:** Elevated plus-maze activity in alcohol-naive male and female rats at day 17 after exposure to predator odor (PO) stress

	Males		Females	
	Control	PO stress	Control	PO stress
% time open arms	29.1 ± 8.0	19.5 ± 3.3	16.5 ± 6.4	24.2 ± 2.9
% entries open arms	30.4 ± 8.1	23.1 ± 3.3	21.3 ± 7.3	29.5 ± 2.7
Closed arms entries	10.9 ± 1.0	12.2 ± 0.7	9.3 ± 2.2	12.1 ± 0.7

Data presented as mean ± SEM

### Experiment 2: predator odor stress reactivity in rats with a history of alcohol drinking

Timeline of experiment 2 procedure is shown in Fig. 2a. Before exposure to PO stress, pair-housed rats were given intermittent access to alcohol for 5 weeks. There was no significant difference on alcohol consumption over the weeks between male and female rats (Fig. 2d; *F* (1, 15) = 1.29, *P* = .27). Likewise, we did not find sex difference on BACs analyzed 2 h after the beginning of the last drinking session (Fig. 2d; *t* = .21, *P* = .83). Independent of sex, avoiders exhibited significantly greater avoidance of the PO-paired chamber at 24 h post-exposure (*F* (1, 18) = 28.79, *P* < .0001) relative to non-avoiders (Fig. 2b). There was no significant difference in the magnitude of avoidance between male and female avoiders (Fig. 2b; *t* = .76, *P* = .48). Although the difference in the proportion of avoiders and non-avoiders in male and female rats did not reach statistical significance (*P* = .39) due to sample size (given a 95% confidence level and 80% power, the recommended sample size would be *n* = 95), the proportion of alcohol-drinking males that met the avoider criteria was 27% higher than alcohol-drinking females (Fig. 2c).

Because we did not find significant differences in the ASR between avoider and non-avoider males (95 dB: *t* = 1.06, *P* = .32; 105 dB: *t* = .50, *P* = .63; 115 dB: *t* = .77, *P* = .46) nor between avoider and non-avoider females (95 dB: *t* = .72, *P* = .49; 105 dB: *t* = .42, *P* = .68; 115 dB: *t* = .06, *P* = .95), animals exposed to bobcat urine were pooled into a group designated PO stress and compared to unstressed controls, segregated by sex. To determine whether PO stress affected startle reactivity differently in male and female rats with a history of alcohol drinking, ASR data were analyzed for each decibel. At 95 dB (Fig. 2e), we found a significant sex × stress interaction effect (*F* (1, 29) = 7.02, *P* = .01). Tukey’s post hoc comparison revealed that PO stressed males exhibited higher startle reactivity than control males (*P* = .03). At 105 dB (Fig. 2f), we found a significant sex × stress interaction effect (*F* (1, 29) = 5.94, *P* = .02). Tukey’s post hoc comparisons revealed that PO stressed males exhibited higher startle reactivity relative to control males (*P* = .04) and also relative to PO stressed females (*P* = .004). At 115 dB (Fig. 2g), we found a significant main effect of sex (*F* (1, 29) = 5.23, *P* = .03) on ASR.

At day 17 post-stress, rats were tested for locomotor activity and anxiety-like behavior in the EPM. We did not find significant differences between avoider and non-avoider males nor between avoider and non-avoider females on percent time in the open arms (males: *t* = .23, *P* = .82; females: *t* = .63, *P* = .55), percent entries in the open arms (males: *t* = .44, *P* = .67; females: *t* = .50, *P* = .63), and closed arm entries (males: *t* = .23, *P* = .82; females: *t* = .62, *P* = .55); thus, animals exposed to bobcat urine were pooled into a group designated PO stress and compared to unstressed controls, segregated by sex. Regardless of stress condition, females with a history of alcohol drinking spent more time in the open arms of the EPM relative to males (Table 2; *F* (1, 30) = 11.86, *P* = .002). Similarly, alcohol-drinking females made more entries in the open arms (Table 2; *F* (1, 30) = 10.17, *P* = .003) and in the closed arm of the EPM relative to males (Table 2; *F* (1, 30) = 4.96, *P* = .03).

**Table 2:** Elevated plus-maze activity in alcohol-drinking male and female rats at day 17 after exposure to predator odor (PO) stress

	Males		Females*	
	Control	PO stress	Control	PO stress
% time open arms	12.5 ± 6.9	13.9 ± 3.1	22.0 ± 6.2	39.2 ± 5.7
% entries open arms	17.5 ± 8.4	21.0 ± 4.4	31.1 ± 6.7	38.3 ± 1.7
Closed arms entries	10.0 ± 1.1	10.9 ± 0.8	12.5 ± 1.5	12.6 ± 0.7

Data presented as mean ± SEM. Regardless of stress condition, females spent more time in the open arms and made more entries into the open and closed arms of the EPM relative to males

*P < .05

### Experiment 3: predator odor stress effects on plasma corticosterone response

The timeline of experiment 3 procedure is shown in Fig. 3a. PO stress produced sexually dimorphic changes in circulating corticosterone levels (Fig. 3b). A two-way repeated measure ANOVA yielded a significant main effect of sex (*F* (1, 9) = 27.21, *P* = .0006), time (*F* (4, 34) = 11.83, *P* < .0001), and a sex × time interaction effect (*F* (4, 34) = 4.65, *P* = .004) on plasma corticosterone response following exposure to bobcat urine. Tukey’s post hoc comparison revealed that corticosterone levels were significantly higher in females relative to males immediately after PO exposure (*P* = .0002), 30 min (*P* < .0001), and 60 min post-stress (*P* = .012). Moreover, in females, corticosterone levels immediately after PO (*P* = .0005) and 30 min post-stress (*P* < .0001) were higher than the baseline.

## Discussion

We report that alcohol-naive male and female rats exposed to PO displayed blunted weight gain 24 h post-stress even though only a subset of stressed animals exhibited avoidance behavior. A similar percentage of alcohol-naive males and females were classified as avoiders after stress, but the proportion of avoiders was higher in alcohol-experienced males relative to females with a history of alcohol drinking. Moreover, PO exposure enhanced startle reactivity in alcohol-experienced males, and females exhibited blunted startle reactivity following odor exposure, regardless of alcohol drinking history. Alcohol-experienced females presented lower anxiety-like behavior and higher general activity in the EPM relative to alcohol-experienced males. Finally, bobcat urine exposure significantly increased circulating corticosterone levels in females immediately after stress until 60 min post-stress compared with males.

In the present study, pair-housed male and female rats consumed comparable amounts of alcohol and achieved similar BACs 2 h after the last drinking session. Thus, sex differences in post-stress startle reactivity are likely not attributable to different levels of alcohol consumption. It is worth mentioning that our animals were pair-housed throughout the experiment, whereas in most studies using the 2-bottle choice protocol, rats were housed individually [33, 36]. While our housing condition does not allow direct associations between alcohol intake and behavioral outcomes in individual animals, it has the benefit of avoiding potentially confounding stressful effects of social isolation.

In line with previous studies [27, 37, 38], bobcat urine exposure elicited significant avoidance in a subset of animals. A similar proportion of alcohol-naive male and female rats exposed to PO were classified as avoiders (males 42%; females 37%), with similar magnitude of bobcat urine-paired context avoidance 24 h post-stress. In animals with a history of ethanol consumption, a higher percentage of males was classified as avoiders (males 67%; females 40%). Interestingly, a systematic review of the comorbidity between PTSD and alcohol misuse found associations between alcohol consumption and the avoidance/numbing and hyperarousal PTSD symptom clusters [39]. Moreover, our group demonstrated that male avoider rats exhibit persistent increases in alcohol self-administration, and avoidance behavior predicts post-stress escalation of alcohol drinking [27, 38].

Although only a subset of rats exposed to PO displayed avoidance behavior, all stressed animals exhibited signs of physiological stress. Our laboratory has previously reported that both avoider and non-avoider male rats exhibited increased anxiety-like behavior following exposure to PO [40]. The current data build on that work by showing that all alcohol-naive stressed male and female rats displayed blunted weight gain 24 h after PO exposure (we did not evaluate changes in body weight in rats with a history of alcohol drinking). Additionally, male rats exposed to bobcat urine have been shown to exhibit a non-significant general increase in startle reactivity [35]. We confirmed and extended these findings by including females and testing animals with a history of chronic alcohol drinking. Here, when tested 6 days after the last drinking session, only alcohol-experienced males showed stress-induced increase in startle reactivity following bobcat urine exposure.

PO stress reduced startle reactivity in females, regardless of alcohol drinking history. Even though we did not measure baseline ASR to counterbalance the groups before the test session, it is unlikely that random assignment of animals would select for rats with different startle reactivity. Prior work has reported lower startle reactivity in female rats after inescapable tail shock stress and identified the presence of ovarian hormones as an important element of stress-induced startle suppression [41]. Interestingly, startle suppression was demonstrated to be more evident when tail shock was applied during estrus [42]. Contrary to these findings, there was no effect of the estrous cycle on adrenocorticotropic hormone (ACTH) and corticosterone responses in female rats exposed to acute restraint stress [43]. In the present study, we did not investigate the effects of estrous cycle on stress reactivity; therefore, the influence of ovarian hormones on behavioral and endocrine responses associated with PO exposure is still to be determined.

Our group reported that bobcat urine exposure increased anxiety-like behavior in male rats 2 and 5 days post-stress [40]. Here, we show that bobcat urine exposure did not alter anxiety-like behavior at day 17 post-stress in alcohol-naive rats. In animals with a history of alcohol drinking, females exhibited lower anxiety-like behavior than males in the EPM (i.e., increased open arm time and open arm entries), and also higher spontaneous motor activity (i.e., increased closed arm entries). Therefore, we cannot rule out the possibility that changes in open arm exploration were affected by alterations in general activity. Similar increase in exploratory activity levels on the EPM was described in Long-Evans female rats following 6-week intermittent access to 20% ethanol in comparison with males exposed to the same procedure [44]. Thus, the higher activity in the EPM observed in females may reflect sex differences in stress/fear coping strategies rather than reduced anxiety-like behavior. In fact, a study using factor analysis showed that in females, activity, rather than anxiety, emerged as the strongest factor in the EPM [45]. Likewise, females are four times more likely than males to display fear in the form of rapid movements instead of freezing in traditional models of Pavlovian fear conditioning [46].

Corticosterone response was higher in females than males immediately after exposure to PO stress until 60 min post-stress, when it returned to baseline levels. Our group showed that male avoider rats exhibit attenuated corticosterone response immediately following exposure to bobcat urine [40]. Differently from this study, animals used here to evaluate sex differences in the time course of corticosterone response were exposed to bobcat urine in a clean cage instead of in the 3-chamber apparatus; thus, they were not indexed as avoiders or non-avoiders. In line with our findings, female rats exposed to acute restraint stress exhibit higher ACTH and corticosterone responses than males, in addition to significantly higher c-fos mRNA expression in the paraventricular nucleus of the hypothalamus [43]. Conversely, TMT exposure elicits similar increase in circulating corticosterone in both male and female Wistar rats [47], indicating that distinct POs (i.e., natural olfactory stimuli or synthetic olfactory stimulus) can produce different responses. Notably, suppression of ASR following combined cat exposure and saline injection in male rats can be blocked by substituting the injected saline with a glucocorticoid receptor antagonist [48].

Systemically circulating corticosterone is coupled to different carriers, mainly corticosteroid binding globulin (CBG) [49]. These proteins play a critical role in regulating glucocorticoid bioavailability by sequestering it in an inactive complex [50, 51]. In mature female rats, CBG content in the serum is 2.5 times higher than in age-matched males [52] because of estradiol-induced increases in hepatic CBG synthesis [53]. This finding highlights the need for future studies aimed at evaluating sex differences in stress reactivity to test levels of bioavailable free corticosterone to determine the biological significance of altered corticosterone levels.

## Conclusions

We report robust sex differences in behavioral and endocrine responses to bobcat urine exposure in adult Wistar rats. Males and females exposed to PO displayed blunted weight gain 24 h post-stress, but only a subset of stressed animals exhibited avoidance behavior. Male rats with a history of chronic moderate alcohol drinking exhibited increased traumatic stress reactivity relative to alcohol-experienced females. Regardless of alcohol drinking history, stressed females exhibited blunted startle reactivity compared with unstressed control females and stressed males. Finally, females presented enhanced corticosterone response following PO stress.

## Perspectives and significance

Sex differences in traumatic stress responses are among the most widely reported phenomena in epidemiological and clinical studies. To the best of our knowledge, the findings reported here are the first to provide evidence that a history of chronic moderate alcohol drinking differentially modulates PO stress reactivity in male and female rats. Our data support the notion that females rather respond differently to trauma and open doors for future work aimed at testing the neurobiology underlying sex differences in traumatic stress reactivity.

## Data Availability

All data are available from the corresponding author upon request.
